# Mobile health: solution or a threat?

**DOI:** 10.1007/s12471-018-1206-1

**Published:** 2018-11-30

**Authors:** R. G. Tieleman, M. E. W. Hemels

**Affiliations:** 1 Department of Cardiology, Martini Hospital, Groningen, The Netherlands; 2 Department of Cardiology, University Medical Center Groningen, University of Groningen, Groningen, The Netherlands; 3 Department of Cardiology, Rijnstate Hospital, Arnhem, The Netherlands; 4 Department of Cardiology, Radboud University Medical Center, Radboud University, Nijmegen, The Netherlands

With the ageing of the population, there is an increasing demand on the healthcare budget.

Mobile health (m-Health) is suggested by many as a possible solution to manage the costs of care [1, 2]. On the other hand, the ease of use and wide availability of m‑Health solutions may increase the workload for professionals, by creating data overload. Automated analysis of data by reliable algorithms is therefore crucial to reduce this workload.

In this issue of the journal, Selder and colleagues describe the results of a study analysing the performance of the Kardia Mobile algorithm as used in the Hartwacht Arrhythmia program [3]. In this program, arrhythmia patients visiting a cardiology outpatient clinic can participate by using an AliveCor Kardia Mobile monitor, which can be used in combination with a smartphone to record a one-lead electrocardiogram (ECG) in case of symptoms. The ECG recording is classified by a smartphone app into four diagnostic categories and subsequently sent to the cardiologist for analysis. In this study, classification of the one-lead ECG by the app was compared with manual classification of the ECG rhythm strip by the cardiologist, which was considered the gold standard. The main findings of the study are that the results of the algorithm are not sufficient for clinical purposes, and that at present manual ECG analysis is needed to reliably diagnose the rhythm. In our opinion, this is a very important finding.

As the authors point out, despite a good sensitivity and specificity of the Kardia device, positive and negative predictive values largely depend on the prevalence and the a priori likelihood of the arrhythmia in the population tested. As an example, if we were to try to find atrial fibrillation in a paediatric clinic, this would only result in false-positive ECGs and a very poor positive predictive value, because atrial fibrillation is exceptionally rare in children. On the other hand, for the same reason, the negative predictive value in this population will be very high.

The present study was performed in a population where all the patients had symptoms possibly related to an arrhythmia and, in fact, most patients had a clinical diagnosis of supraventricular or ventricular arrhythmias in their history. In this respect, we expect a high positive predictive value for diagnosing arrhythmias such as atrial fibrillation, considering a specificity of the AliveCor algorithm of between 71 and 99% [4]. Indeed, the automatic algorithm had a relatively high positive predictive value of 80% for detection of atrial fibrillation. However, this still means an incorrect diagnosis in 20% of the positive results, which is not good enough to solely rely on the automatic diagnosis, and manual interpretation of all positive ECGs is warranted. Furthermore, almost 20% of the ECGs were either unclassified or unreadable by the algorithm, which also necessitates additional manual review by the cardiologist. The algorithm detected sinus rhythm in 59% of the patients, with a positive predictive value of 96% (when including extrasystole, 90% when excluding extrasystole). This can be considered good enough for automatic reassurance of the patient, although it may be argued that this is still incorrect in 1 out of 25 patients (or 1 out of 10 when excluding extrasystole). In the other 41%, the algorithm either diagnosed atrial fibrillation, an unclassified arrhythmia or considered the recording unreadable. Because of the low positive predictive value of this automatic classification, the authors correctly conclude that manual interpretation of all the ECGs that are not classified as sinus rhythm is warranted, despite the high-risk population that was studied. For the 233 patients, this resulted in 2,453 ECGs to be evaluated by the cardiologist.

Therefore, this m‑Health tool in its present state will not decrease the workload of the cardiologist, and may even increase the demands on the staff. On the other hand, patients may be treated at home instead of coming to the hospital, and from the patients’ point of view, use of the Hartwacht Arrhythmia program may increase patient satisfaction and quality of life for various reasons. In addition, it may also be cost-effective, but that was not part of this study and should be further investigated.

When programs like this expand to lower risk populations, for example with the wide availability of smartwatches with ECG recording in the general population, the number of false-positive results will increase even more, putting more demands on health resources instead of less. It is even possible that the increase in false-positive signs of arrhythmias in a healthy population may actually have a detrimental effect on quality of life, at least in this automated form of detection. Therefore, taking the current knowledge of incidence and prevalence of different arrhythmias into account, the added value and cost-effectiveness of m‑Health solutions is largely dependent on the accuracy of the automated algorithms and the population in which it is used. When done wrong, m‑Health may increase the demand on the budget, contrary to common belief.
